# Comparative inhalation toxicity of multi-wall carbon nanotubes, graphene, graphite nanoplatelets and low surface carbon black

**DOI:** 10.1186/1743-8977-10-23

**Published:** 2013-06-17

**Authors:** Lan Ma-Hock, Volker Strauss, Silke Treumann, Karin Küttler, Wendel Wohlleben, Thomas Hofmann, Sibylle Gröters, Karin Wiench, Bennard van Ravenzwaay, Robert Landsiedel

**Affiliations:** 1 Experimental Toxicology and Ecology, BASF SE, 67056 Ludwigshafen, Germany; 2 Material Physics, BASF SE, 67056 Ludwigshafen, Germany; 3 Product Safety, BASF SE, 67056 Ludwigshafen, Germany

**Keywords:** Inhalation toxicity, Graphene, Multi-wall carbon nanotubes, Graphite nanoplatelets, Carbon black

## Abstract

**Background:**

Carbon nanotubes, graphene, graphite nanoplatelets and carbon black are seemingly chemically identical carbon-based nano-materials with broad technological applications. Carbon nanotubes and carbon black possess different inhalation toxicities, whereas little is known about graphene and graphite nanoplatelets.

**Methods:**

In order to compare the inhalation toxicity of the mentioned carbon-based nanomaterials, male Wistar rats were exposed head-nose to atmospheres of the respective materials for 6 hours per day on 5 consecutive days. Target concentrations were 0.1, 0.5, or 2.5 mg/m^3^ for multi-wall carbon nanotubes and 0.5, 2.5, or 10 mg/m^3^ for graphene, graphite nanoplatelets and low-surface carbon black. Toxicity was determined after end of exposure and after three-week recovery using broncho-alveolar lavage fluid and microscopic examinations of the entire respiratory tract.

**Results:**

No adverse effects were observed after inhalation exposure to 10 mg/m^3^ graphite nanoplatelets or relatively low specific surface area carbon black. Increases of lavage markers indicative for inflammatory processes started at exposure concentration of 0.5 mg/m^3^ for multi-wall carbon nanotubes and 10 mg/m^3^ for graphene. Consistent with the changes in lavage fluid, microgranulomas were observed at 2.5 mg/m^3^ multi-wall carbon nanotubes and 10 mg/m^3^ graphene. In order to evaluate volumetric loading of the lung as the key parameter driving the toxicity, deposited particle volume was calculated, taking into account different methods to determine the agglomerate density. However, the calculated volumetric load did not correlate to the toxicity, nor did the particle surface burden of the lung.

**Conclusions:**

The inhalation toxicity of the investigated carbon-based materials is likely to be a complex interaction of several parameters. Until the properties which govern the toxicity are identified, testing by short-term inhalation is the best option to identify hazardous properties in order to avoid unsafe applications or select safer alternatives for a given application.

## Background

Engineered nanomaterials have unique physicochemical properties and are commonly used in industrial applications like microelectronics, lightweight materials, functional textiles, or coatings to achieve distinctly improved properties. Carbon Black is the conventional filler in rubber to achieve the grip and mechanical stability of tires and for their black color. Carbon Black is soot that consists of primary particles with diameters smaller than 100 nm in all three dimensions. It has been manufactured for decades in quantities in the range of megatons per year [1]. One of the most significant examples of novel engineered nanomaterials is carbon nanotubes (CNT), which are one-dimensional seamless tubes of single-walled (SWCNT) or multi-walled (MWCNT) carbon sheets. They possess high tensile strength and electrical conductivity, and are technically valuable because of their much lower specific density compared to metals or inorganic semiconductors [2]. Graphene is a monolayer of carbon atoms arranged in a two-dimensional honeycomb network. This definition is not restricted to a material consisting exclusively of single-layer material but, like in many publications and as used by most commercial providers, rather denotes a bulk material, which is generally a mixture of single and multiple layer material. Graphene has spurred the imagination of engineers and scientists with properties that may in part replace CNTs (*e.g.* for conductive thin films), or replace conventional two-dimensional fillers such as clays (*e.g.* for barrier properties), promoting development of some unique devices, *e.g.* in photonics, supercapacitors, or bioapplications [3-5]. Likewise, graphite nanoplatelets have attracted interest for a variety of applications due to properties which differ to those of graphite [6]. Increasing use of carbon-based nanomaterials did raise concern about potential toxic effects. The implications of a nanomaterials’ dimensionality for their hazard profile have been highlighted recently on the issue of exceptionally high surface to volume ratio of graphene as compared to carbon nanotubes [7].

Although oral and dermal absorption of nanomaterials can in principle occur, inhalation is considered to be the exposure route of highest concern in humans. Accordingly animal experiments have been performed using intratracheal instillation, pharyngeal aspiration, and inhalation exposure to nanomaterials.

Among the carbon-based materials, Carbon Black was the most examined in the past. It is known that inhaled nano-size Carbon Black particles (Printex 90) cause inflammation and type II epithelial hyperplasia in rats [8]. Exposure of female rats to 6 and 12.2 mg/m^3^ Carbon Black (Printex 90, particles size 15 nm, surface area 213 m^2^/g) for two years led to increased tumor incidence in the lung [9]. In contrast to the high surface area Carbon Black, low surface area Carbon Black (37 m^2^/g) induced less severe inflammatory changes on a mass-based dose [8]. However, in a further carcinogenicity study Elftex 12 (surface area 43 m^2^/g) caused concentration-related increased lung weight, polymorphonuclear neutrophils and biochemical changes of broncho-alveolar lavage fluid (BALF), and increased incidence of adenomas and adenocarcinomas in rats [10-12].

Intratracheal administration of SWCNTs or MWCNTs caused pulmonary inflammation, characterized by changes in cellularity and enzyme activities of BALF and associated microscopic findings like macrophage infiltration, epitheloid granulomas, and fibrotic changes in mice and rats [13-20]. Only mild and transient effects without evidence of chronic inflammation were observed after intratracheal instillation of individually dispersed MWCNTs [21] or nanoscale dispersed SWCNTs [19]. The needle shaped structure of some MWCNTs is similar to asbestos, and asbestos-like pathological changes were observed after intraperitoneal injection of long MWCNTs. This fact has raised concern that chronic exposure may lead to mesothelioma [22]. Mesotheliomas were observed after intraperitoneal administration of MWCNTs in p53^+/−^ mice [23]. In another study, asbestos, but not MWCNTs produced mesotheliomas after intraperitoneal injection after two years in rats [24]. However, the MWCNTs used by Muller et al. [24] were significantly shorter than the one in Poland’s study [22], which may have been one reason for the absence of carcinogenic response.

In contrast to intratracheal instillation, neither lung tissue damage nor inflammation was observed in two short-term inhalation studies with MWCNTs in mice [17,25] and after inhalation exposure to well-dispersed SWCNT in rats [26]. However, histopathological lesions in the upper and lower respiratory tract were observed after inhalation exposure of rats to MWCNT for three months. Examinations of BALF revealed inflammatory changes, as shown by distinctly increased numbers of polymorphonuclear neutrophils [27,28].

In comparison to carbon nanotubes data on toxicity of graphene after intratracheal instillation are limited. Increased cell counts, protein and interleukin (IL)-6 concentrations were observed after intratracheal instillation of aggregated and nanoscale dispersed graphene [29]; the changes were more marked after application of aggregated graphene. Schinwald et al. [7] administered graphene by pharyngeal aspiration or intrapleural injection in mice and lavaged lung and pleural space. Increased numbers of polymorphonuclear neutrophils and increased levels of pro-inflammatory cytokines (monocyte chemoattractant protein (MCP)-1, macrophage inflammatory protein (MIP)-1R, MIP-1, IL-1β) were present in both the broncho-alveolar and pleural lavage fluid. These changes were not observed after administration of Carbon Black under the same conditions. Inhalation toxicity studies with graphene or graphite nanoplatelets have not been reported yet.

The above described studies indicate variable toxicological responses to the different carbon-based materials. The present short-term inhalation study has been conducted to compare the effects of different carbon-based materials with the same chemistry but different structure. Since the materials consist mainly of agglomerated primary particles, the different forms (tubular, spherical, leaf-shaped, platelet) were expected to contribute to a lesser extent to toxicity compared to the parameters surface, volume, mass, or surface reactivity. Correlation of toxicological parameters was made with different physico-chemical properties to identify the triggering parameter for toxicity in order to assist in the selection of safer materials at an early development stage. This short-term inhalation study design was optimized for the specific properties of nanomaterials by incorporation of additional endpoints like measurement of pro-inflammatory cytokines in BALF. Selection of concentrations was based on available inhalation toxicity data and on the current occupational exposure limit for nuisance dust in Germany (until 2011: 1.5 mg/m^3^, since 2011: 300 μg/m^3^ for granular biopersistent materials) [30].

## Results

### Characterization of test materials

**Figure 1:**
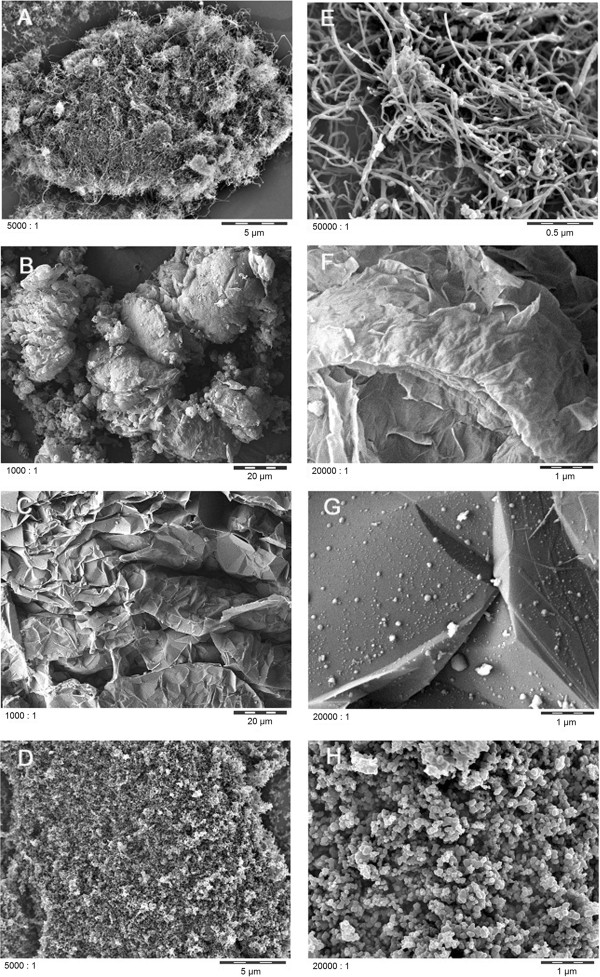
**Scanning electron microscopy images of the test materials. A**-**D**: agglomerate scale, **E**-**H**: primary structure scale. **A**, **E**: MWCNT, **B**, **F**: graphene, **C**, **G**: graphite nanoplatelets, **D**, **H**: Carbon Black. Note the same scale for MWCNT and Carbon Black, and slightly different set of scales for graphite nanoplatelets and graphene, in order to point to their individual characteristics.

**Figure 2:**
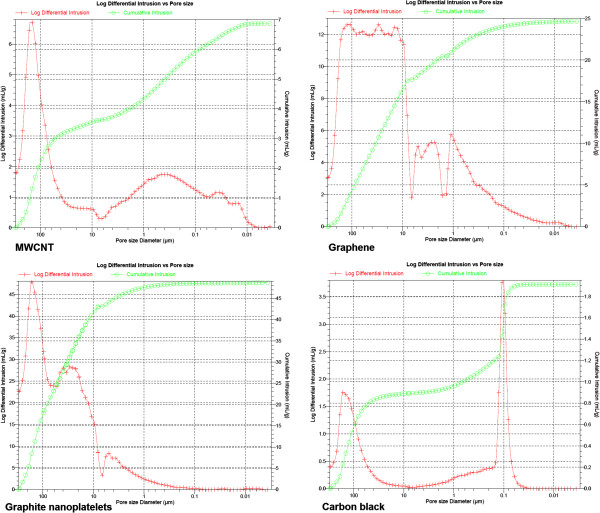
Diagrams of mercury intrusion porosimetry experiments with MWCNT, graphene, graphite nanoplatelets and carbon black.

The combination of morphological imaging by scanning electron microscopy (SEM) (Figure 1) with quantified porosimetry by Hg intrusion highlights the drastically different mesoscopic structures. The specific MWCNT investigated here revealed the expected primary structure of 15 nm outer diameter fibers, but showed the formation of fibrilles at the intermediate structural range between 100 nm and 1 μm (Figure 2), and large agglomerates of tens to hundreds of μm (Figures 1 and 2). The fibrillar intermediate structure makes the agglomerates rather spongy in comparison to other MWCNT materials such as NM400 from the OECD sponsorship program, which has a similar outer diameter of 9.5 nm. In NM400 this intermediate structure is absent, and agglomerates are formed from the individual nanotubes. Carbon Black consisted of globular shaped particles with a primary size of 50 to 100 nm, no intermediates structure up to agglomerates of tens to hundreds of μm (Figures 1 and 2). The expected three-dimensional nanostructure of Carbon Black is clearly confirmed by the SEM images, as well as the one-dimensional primary structure of MWCNT. The two other nanomaterials are obtained by (partial) exfoliation of graphite and are nominally two-dimensional Carbon, but differ drastically at all length scales. Even the material that we designate as graphene does not deserve this name in a strict sense, because it consists of thin multilayers, not monolayers. The smallest observable structural peak in the pore size distribution at 9 nm may be indicative of the dominating thickness range (Figure 2), with lateral sheet dimensions of many μm (Figure 1). The contribution of pore sizes below 100 nm evidences the contrasting structure of graphite nanoplatelets, which is clearly two-dimensional (Figure 1C) but has only a vanishing contribution of pores below 100 nm (Figure 2), and instead has a primary structure of platelets around and above 100 nm (Figure 1G). The Xray diffraction with sharp peaks (Rietveld-Scherrer evaluation of 19 nm crystallite size) further supports the existence of comparably thick platelets. The agglomerate density was read from the cumulative volume of pores with sizes below the approximate diameter of agglomerates (taking 1 μm as cut-off), and is an indicator for the volume occupied at equal mass after lung deposition.

Carbon Black and MWCNT were of rather high carbon purity above 98%. By X-ray photoelectron spectroscopy (XPS) with its information depth of 10 nm (enough to reach the core of the MWCNT), remaining metals from the catalyst used for MWCNT production were not observed.

Graphite nanoplatelets and graphene were of lower carbon purity: Sulfur impurities at different oxidation states from the intercalating acids used for exfoliation, and oxygen from partial oxidation of the carbon material. The structural purity was further assessed by Raman spectroscopy [31]. The Raman spectrum of Carbon Black matches the soot reference, whereas the Raman spectrum of graphite nanoplatelets matches the bulk graphite reference. The G-band is shifted to 1590 cm^-1^ only for the Carbon Black, which also has a very low Raman intensity, confirming a dominantly amorphous material. Although both two-dimensional carbon materials share a dominance of the G band over the G’ band, the G’ band is found at 2663 cm^-1^ for graphene, but at 2702 cm^-1^ for graphite nanoplatelets, and the relative intensity of D-band *versus* G-band decreases from 1.40 ± 0.05 (graphene) to 0.01 ± 0.01 (graphite nanoplatelets). Together with the SEM results (Figure 1), the Raman results thus confirm the strong structural difference of thicker low-defect graphite nanoplatelets against few-layer graphene with vanishing monolayer content and only little nm average distance between two defects [31]. Apart from sulfur and oxygen, the content of thicker three-dimensional carbon structures must also be considered an impurity for a nanomaterial that is engineered to be two-dimensional. The purity of graphite nanoplatelets and graphene is thus at about 85%.

A comprehensive list of physico-chemical characteristics is given in Table 1.

**Table 1:** Physico-chemical parameters of the test materials

**Property**	**MWCNT**		**Graphene**		**Graphite nanoplatelets**		**Carbon black**	
Representative images (SEM)	See Figure 1		See Figure 1		See Figure 1		See Figure 1	
Particle size Distribution (SEM)primary structure	15 nm, fiber shape		Up to 10,000 nm diameter, flakes		Up to 30,000 nm diameter, flakes		50-100 nm, globular	
State of agglomeration	~20,000 nm, bundles		~40,000 nm, ‘crumpled napkin’		>50,000, ‘crumpled cardboard’		>20,000 nm	
Crystallite size (XRD)	Dominatingly amorphous in XRD		Dominatingly amorphous in XRD		Contains amorphous material, but has sharp diffraction peak corresponding to 19 nm crystallite size		Dominatingly amorphous in XRD	
Crystalline phase (XRD)	Graphite, C hexagonal with modified lattice const.		Possibly graphite, sync –C– hexagonal or carbon –C– hexagonal, or carbon –C– cubic or carbon - C		Graphite, sync– C - hexagonal		Graphite, C hexagonal with modified lattice const.	
Pore sizes (Hg intrusion)	25 nm, 400 nm, 150,000 nm		9 nm, 100 nm, 40,000 nm		6,000 nm, 30,000 nm, 150,000 nm		100 nm, 150,000 nm	
Surface area (Hg) [mg/m^2^]	161		131		74		32	
Apparent density (g/mL)	0.15		0.02		0.04		0.52	
Agglomerate density (g/mL)	0.39		0.29		0.89		1.05	
Surface chemistry (%) (XPS)	C	99	C	84.1	C	84.3	C	98
	O	1	O	8.8	O	9.0	O	1
			S	5.4	S	1.7	S	1
			Na	0.6	Na	3.0	Cl	<<1
			Si	0.4	Ca	1.5		
			Cl	0.6	Si	0.6		
Raman peak positions (cm^-1^) (Laser spectroscopy)	G	1574		1574		1573		1590
	D	1338		1338		1348		1348
	D/G	1.16 ± 0.04		1.40 ± 0.05		0.01 ± 0.01		-
Dispersability (by AUC)	agglomerated		agglomerated		agglomerated		agglomerated	
In water	<0.01 wt% below 100 nm		<0.01% below 100 nm		<0.01% below 100 nm		1,920 nm	
In DMEM/FCS	agglomerated at several μm						Dispersed below 100 nm	
Purity (combined assessment from XRD, ICP-MS, XPS data)	99%		< 85%		< 85%		98%	
Identified impurities	None significant		Sulfur impurity (presumably from acid intercalation before exfoliation)		Na-, Ca, Si-containing minerals		None significant	
			3D-Graphite (not 2D-Graphene) impurities		3D-Graphite (not 2D-Graphene) impurities			

### Characterization of the test atmosphere

Results of gravimetric determination of aerosol concentrations and particle size distribution are summarized in Table 2.

**Table 2:** Results of gravimetric determination of test atmosphere concentration and particle size distribution

**MWCNT**				
Target concentration (mg/m^3^)		0.1	0.5	2.5
Measured concentration (mg/m^3^ ± SD)		0.15 ± 0.05	0.57 ± 0.10	2.86 ± 0.82
MMAD (μm)		1.1/1.7	1.3/0.9	2.0/1.2
GSD		3.7/3.0	3.7/3.8	3.0/3.5
OPC	Count median diameter (μm)	0.494	0.499	0.533
	Count concentration (N/cm^3^)	70	405	724
SMPS	Count median diameter (μm)	0.145	0.096	0.127
	Count concentration (N/cm^3^)	1072	5636	4410
Graphene				
Target concentration (mg/m^3^)		0.5	2.5	10.0
Measured concentration (mg/m^3^ ± SD)		0.54 ± 0.04	3.05 ± 1.05	10.1 ± 4.5
MMAD (μm)*		≤ 0.4	< 0.4	< 0.4
OPC	Count median diameter (μm)	0.473	0.977	0.656
	Count concentration (N/cm^3^)	57	191	343
SMPS	Count median diameter (μm)	0.102	0.436	0.508
	Count concentration (N/cm^3^)	269	98	226
Graphite nanoplatelets				
Target concentration (mg/m^3^)		0.5	2.5	10
Measured concentration (mg/m^3^ ± SD)		0.46 ± 0.11	2.08 ± 0.33	10.27 ± 1.44
MMAD (μm)		1.7/1.6	2.1/2.3	2.7/3.4
GSD		2.4/2.2	2.8/2.5	2.7/2.5
OPC	Count median diameter (μm)	0.373	0.373	0.360
	Count concentration (N/cm^3^)	458	1828	7401
SMPS	Count median diameter (μm)	0.123	0.146	0.148
	Count concentration (N/cm^3^)	2819	9893	35606
Carbon Black				
Target concentration (mg/m^3^)		0.5	2.5	10
Measured concentration (mg/m^3^ ± SD)		0.5 ± 0.1	2.5 ± 0.2	10.9 ± 1.5
MMAD (μm)/GSD		0.9/0.8	0.6/0.7	0.9/0.6
GSD		2.7/2.1	3.6/3.1	4.5/2.7
OPC	Count median diameter (μm)	0.40	0.38	0.38
	Count concentration (N/cm^3^)	1231	7418	35829
SMPS	Count median diameter (μm)	0.24	0.22	0.23
	Count concentration (N/cm^3^)	21138	122097	371078

*OPC* Optical Particle Counter, *SMPS* Scanning Mobility Particle Sizer.

* GSD could not be determined (majority below the smallest impactor stage).

Measured concentrations of aerosols were close to the target concentrations.

The mass median aerodynamic diameters (MMAD) of the particles in the aerosols were small enough to reach the lower respiratory tract of rats (the particles were respirable for rats).

**Figure 3:**
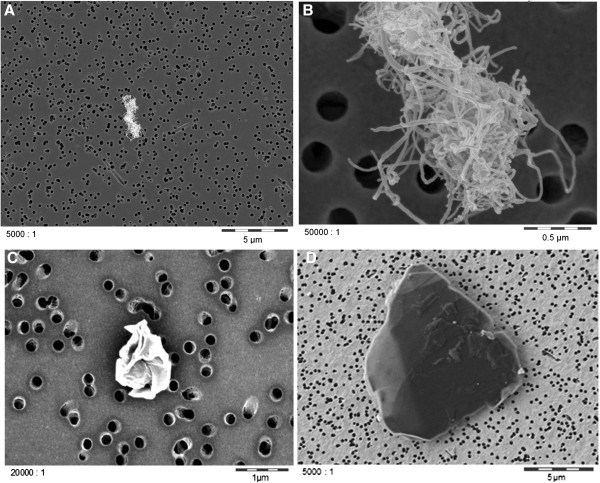
**Scanning electron images of representative test atmosphere samples. A**, **B**: MWCNT, **C**: graphene, **D**: graphite nanoplatelets.

Scanning electron microscopic evaluation of the test atmospheres collected on gold-coated capillary filters is shown in Figure 3. MWCNTs showed a wool-like structure, which was comparable to the appearance of the material before aerosol generation. Graphene particles were considerably smaller than those of graphite nanoplatelets, which matched with the results obtained with the raw materials.

### Clinical observations

Inhalation of MWCNT, graphene, graphite nanoplatelets, or Carbon Black did not cause any adverse clinical signs. The body weight development of exposed rats was comparable to control animals.

### Hematology and acute phase proteins in serum

In the studies of all four compounds no toxicological relevant effects were observed regarding hematology and the α_2_-macroglobulin as well as haptoglobin levels. If inflammatory response occurred (with MWCNT and graphene) they were restricted to the lungs.

### Broncho-alveolar lavage and lung tissue homogenates

In case of mediators, only parameters which were altered by exposure to the test substances are shown in the tables.

#### MWCNT

Differences of parameters measured in BALF and lung tissue homogenates when compared to the controls are shown in Tables 3, 4, 5 and 6.

**Table 3:** Cytology parameters in BALF after exposure to MWCNT

	**Study day 7**			**Study day 28**		
Concentration [mg/m^3^]	0.1	0.5	2.5	0.1	0.5	2.5
Total Cells	1.1	1.4	3.6^**^	1.1	1.0	2.1^*^
Polymorphonuclear Neutrophils	1.4	72.8^**^	436.6^**^	4.9	8.8	343.8^**^
Lymphocytes	0.9	5.6	41.3^**^	1.4	3.9	9.6^**^
Macrophages	1.1	0.8	0.3^*^	1.1	0.9	1.1

* Statistically significant, p < 0.05; ** statistically significant, p < 0.01.

Differences are expressed as x-fold ratios compared to the concurrent control group.

**Table 4:** Protein concentration and enzyme activities in BALF after exposure to MWCNT

	**Study day 7**			**Study day 28**		
Concentration [mg/m^3^]	0.1	0.5	2.5	0.1	0.5	2.5
Total Protein	1.1	1.5	3.5^**^	1.1	1.0	1.7
GGT	1.4	5.1^**^	7.4^**^	0.8	3.5^*^	9.5^**^
LDH	1.3	2.0^**^	5.2^**^	1.2	1.1	2.4^**^
ALP	1.3	2.3^*^	3.8^**^	0.9	0.9	1.5^*^
NAG	1.0	1.4	2.8^**^	0.9	1.3	2.1

* Statistically significant, p < 0.05; ** statistically significant, p < 0.01.

Differences are expressed as x-fold ratios compared to the concurrent control group.

**Table 5:** Mediators in BALF after exposure to MWCNT

	**Study day 7**			**Study day 28**		
Concentration [mg/m^3^]	0.1	0.5	2.5	0.1	0.5	2.5
β2-Microglobulin	1.1	1.6^**^	2.8^**^	1.1	1.2	2.2^**^
CINC-1/IL-8	1.0	4.6^**^	13.3^**^	1.2	2.1^*^	10.5^**^
Clusterin	1.5	1.9^*^	4.4^**^	1.0	1.1	2.2^*^
GCP-2	0.9	8.1^**^	19.9^**^	1.6	4.0^*^	36.2^**^
KC/GROα	1.5	6.3^*^	36.9^**^	0.7	1.6	20.8^**^
MCP-1	1.0	2.3^**^	15.8^**^	1.5	1.6^*^	13.5^**^
MCP-3	1.0	2.5^**^	22.3^**^	1.6	1.7^*^	15.9^**^
M-CSF	1.4	4.3^**^	9.6^**^	1.1	1.7^*^	3.7^**^
MDC	1.2	6.1	50.8^**^	1.1	1.8	20.6^**^
MIP-1β	0.7	1.2	3.1^**^	1.6	1.5	4.1^**^
MIP-2	1.1	2.6^**^	4.5^**^	1.5	1.7^*^	5.4^**^
MPO	1.6	29.5^*^	193.3^**^	1.4	1.9	65.5^**^
VEGF	1.1	2.1^*^	5.0^**^	1.0	1.0	1.6

* Statistically significant, p < 0.05; ** statistically significant, p < 0.01.

Differences are expressed as x-fold ratios compared to the concurrent control group.

**Table 6:** Mediator levels in lung tissue after exposure to MWCNT

	**Study day 7**			**Study day 28**		
Concentration [mg/m^3^]	0.1	0.5	2.5	0.1	0.5	2.5
CINC-1/IL-8	1.2	3.0^**^	6.8^**^	1.0	1.9^*^	4.6^**^
KC/GROα	2.0	9.7^**^	21.3^**^	1.0	3.3^*^	18.8^**^
MCP-1	1.1	1.2	3.1^**^	0.8	0.9	1.8^*^
MCP-3	0.9	1.0	2.2^**^	0.9	1.0	1.7^*^
M-CSF	1.2	1.5^*^	2.3^**^	1.1	1.2^*^	1.6^**^
MDC	1.4	2.1	8.8^**^	0.9	1.0	2.6^*^
MIP-2	1.2	2.5^**^	5.1^**^	0.9	1.4^*^	4.2^**^
MPO	1.3	1.3	2.2^**^	1.1	1.0	1.3
NGAL	1.4^*^	2.2^**^	4.7^**^	1.1	1.2	2.9^**^
Osteopontin	1.1	1.6	3.1^**^	0.6	0.9	2.9^**^

* Statistically significant, p < 0.05; ** statistically significant, p < 0.01.

Differences are expressed as x-fold ratios compared to the concurrent control group.

On study day 7, a significant increase of the total cell counts was found at 2.5 mg/m^3^. This increase was mainly due to high polymorphonuclear neutrophil counts and to a lesser degree by an increase of the lymphocyte counts. In contrast, macrophage counts were significantly decreased at 2.5 mg/m^3^. Significantly increased polymorphonuclear neutrophil counts were also present at 0.5 mg/m^3^.

On study day 28, cell counts were lower, but total cell count as well as polymorphonuclear neutrophil and lymphocyte counts were still increased at 2.5 mg/m^3^.

On study day 7, total protein levels and enzyme activities were markedly increased at 2.5 mg/m^3^. γ-glutamyltranspeptidase (GGT), lactate dehydrogenase (LDH) and alkaline phosphatase (ALP) activities were also dose-dependently increased at 0.5 mg/m^3^.

On study day 28, total protein levels at 2.5 mg/m^3^ were higher, but no longer statistically significantly increased. However, activities of LDH, GGT and ALP were still significantly increased. The higher GGT activity at 0.5 mg/m^3^ was in the range of the historical controls, and therefore not considered to be toxicologically relevant.

Levels of 13 of the 69 measured mediators listed in Table 5 were above the detection limit and statistically significantly increased at 2.5 mg/m^3^. On study day 7, most of the parameters were also increased at 0.5 mg/m^3^.

On study day 28, mediator concentrations were generally lower compared to study day 7 with the exception of the granulocyte chemotactic peptide (GCP)-2 level, but were still statistically significantly increased compared to the controls at 2.5 mg/m^3^. Slightly increased levels of GCP-2, MCP-1, MCP-3, macrophage colony stimulating factor (M-CSF) and MIP-2 were present also at 0.5 mg/m^3^.

On study day 7, levels of 10 mediators listed in Table 6 were above the detection limit and were significantly increased at 2.5 mg/m^3^. Most mediator levels, which were increased in the lung tissue homogenates at 2.5 mg/m^3^ were also increased in BALF. Increases in the BALF were higher compared to those in lung tissue homogenates. MCP-1, MCP-3 and myeloperoxidase (MPO) concentrations were statistically significantly increased in the BALF at 0.5 mg/m^3^, but not in lung tissue samples. The levels of β_2_-Microglobulin, Clusterin, GCP-2, MIP-1β and vascular endothelial growth factor (VEGF) were only increased in BALF samples at 2.5 mg/m^3^. However, two mediator levels (neutrophil gelatinase-associated lipocalin (NGAL) and Osteopontin) were increased in lung tissue homogenates only.

On study day 28, mediator concentrations in the lung tissue homogenates at 2.5 mg/m^3^ were smaller, except growth-related oncogene (KC/GROα) and Osteopontin, but were still significantly increased compared to the controls. Slight increases of the cytokine-induced neutrophil chemoattractant (CINC)-1/IL-8, KC/GROα, M-CSF and MIP-2 levels were already found at 0.5 mg/m^3^.

#### Graphene

Changes in BALF and lung tissue parameters of exposed animals compared to control animals were observed and are summarized in Tables 7, 8, 9, 10.

**Table 7:** Cytology parameters in BALF after exposure to graphene

	**Study day 7**			**Study day 28**		
Concentration [mg/m^3^]	0.5	2.5	10	0.5	2.5	10
Total Cells	0.9	0.7	1.7	1.0	1.3	1.5^**^
Eosinophils	0.5	1.4	8.2^**^	1.7	2.3	0.0
Polymorphonuclear Neutrophils	1.2	7.5^*^	102.8^**^	1.0	3.0^*^	16.5^**^
Lymphocytes	3.0	4.2	33.4^**^	1.9	2.7	5.7^**^
Macrophages	0.8	0.6	0.6	1.0	1.2	1.2

* Statistically significant, p < 0.05; ** statistically significant, p < 0.01.

Differences are expressed as x-fold ratios compared to the concurrent control group.

**Table 8:** Protein concentration and enzyme activities in BALF after exposure to graphene

	**Study day 7**			**Study day 28**		
Concentration [mg/m^3^]	0.5	2.5	10	0.5	2.5	10
Total Protein	1.3	1.2	2.9	1.2	1.4^*^	1.8^**^
GGT	1.0	4.4	10.3^**^	+	+	+
LDH	0.9	1.1	2.8	1.2	1.6^*^	2.2^**^
ALP	1.1	1.5^*^	3.2^**^	1.2	1.0	1.9^**^
NAG	1.0	1.2	1.8	0.8	1.0	1.1

* Statistically significant, p < 0.05; ** statistically significant, p < 0.01.

+: Increased compared to controls (activity below detection limit in controls).

Differences are expressed as x-fold ratios compared to the concurrent control group.

**Table 9:** Mediator levels in BALF after exposure to graphene

	**Study day 7**			**Study day 28**		
Concentration [mg/m^3^]	0.5	2.5	10	0.5	2.5	10
CINC-1/IL-8	1.4	2.3^**^	5.9^**^	1.2^*^	1.7^*^	3.9^**^
MCP-1	2.0	1.5^*^	11.1^**^	1.1	1.7^*^	4.6^**^
Osteopontin	2.3^*^	1.8	13.1^**^	1.8^*^	4.6^**^	18.6^**^

* Statistically significant, p < 0.05; ** statistically significant, p < 0.01.

Differences are expressed as x-fold ratios compared to the concurrent control group.

**Table 10:** Mediator levels in lung tissue after exposure to graphene

	**Study day 7**			**Study day 28**		
Concentration [mg/m^3^]	0.5	2.5	10	0.5	2.5	10
IL-1α	1.2	1.6**	3.0**	1.0	1.4	1.6

** Statistically significant, p < 0.01.

Differences are expressed as x-fold ratios compared to the concurrent control group.

Total cell counts were higher at 10 mg/m^3^ at both time points. Lymphocyte and polymorphonuclear neutrophil counts were increased at 10 mg/m^3^ at both time points, but the changes were considerably more pronounced at study day 7. Additionally, eosinophil counts were increased at 10 mg/m^3^ on study day 7.

Protein levels were increased at 10 mg/m^3^ at both time points. Additionally, increased activities of GGT, LDH, and ALP were observed. N-Acetyl-β-Glucosaminidase (NAG) activities were comparable among groups. Slightly higher, but statistically significant changes in ALP activities occurred at 2.5 mg/m^3^ on study day 7, and in total protein concentration and LDH activities at 2.5 mg/m^3^ on study day 28. However, these effects were minor and therefore not considered to be of toxicological relevance.

Changes of mediators below factor 2 were not considered to be toxicologically relevant. At 2.5 and 10 mg/m^3^, CINC-1/IL-8, and MCP-1 levels were dose-dependently increased at both time points. Osteopontin concentrations were increased at 10 mg/m^3^ on day 7 and day 28 and at 2.5 mg/m^3^ on study day 28.

Increased levels of IL-1α were present on study day 7 in lung tissue homogenates of rats exposed to 10 mg/m^3^.

**Figure 4:**
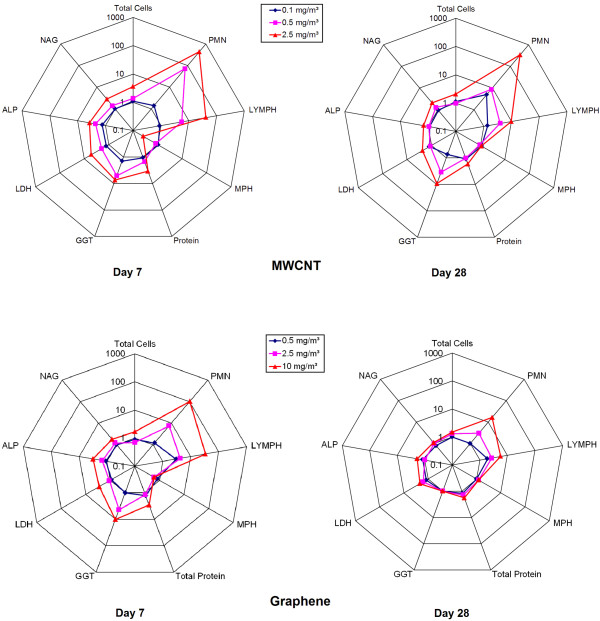
**Comparison of changes in BALF parameters after exposure to MWCNT and graphene.** Changes are shown as x-fold differences compared to controls using a logarithmic scaling.

A comparison of cytology and enzyme changes in BALF after exposure to MWCNT and graphene is shown in Figure 4.

#### Graphite nanoplatelets

On study day 7, approximately 3-fold higher GGT activity was measured in BALF of animals exposed to 10 mg/m^3^ compared to that of control animals. There was no change in any other enzyme activity, and total protein content.

#### Carbon Black

No compound-related effects were observed in rats exposed to Carbon Black.

### Organ weights

Absolute and relative lung weights were increased at 0.5 and 2.5 mg/m^3^ MWCNT on study day 4 by approximately 12%. No compound-related effects on organ weights were observed after inhalation of graphene, graphite nanoplatelets and Carbon Black.

### Macroscopic and microscopic examination

No compound-related macroscopically visible findings were noted at necropsy after exposure to any of the compounds. Microscopically, compound-related adverse effects were observed in rats exposed to MWCNT and graphene but not graphite nanoplatelets and Carbon Black.

#### MWCNT

**Figure 5:**
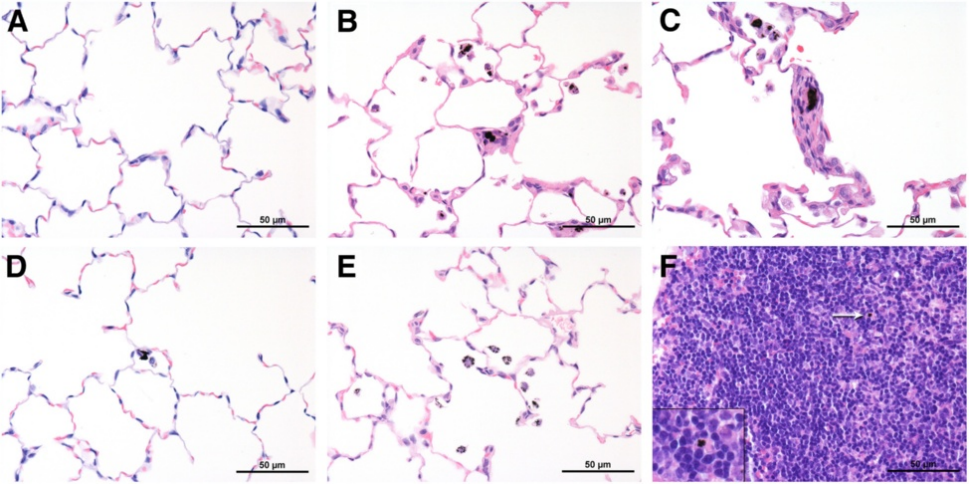
**Microscopic appearance of lungs (A-E) and lymph node (F). A** – Control. **B** – MWCNT, 2.5 mg/m^3^, study day 4: Microgranuloma, particles in the lesion and inside of alveolar macrophages. **C** – Graphene, 10 mg/m^3^, study day 7: Microgranuloma, particles within the lesion. **D** – Graphite nanoplatelets, 10 mg/m^3^, study day 4: Particles inside of alveolar macrophages. **E** – Carbon Black, 10 mg/m^3^, study day 4: Particles inside of alveolar macrophages. **F** – MWCNT, 2.5 mg/m^3^, study day 25: Mediastinal lymph nodes with macrophages containing black particles (arrow).

On study day 4, the lungs of one animal exposed to 2.5 mg/m^3^ revealed intra-septally located microgranulomas (diagnosed as granulomatous inflammation) which were composed mainly of alveolar macrophages. All animals exposed to 2.5 mg/m^3^ showed a diffuse alveolar histiocytosis (single macrophages within the alveolar space distributed all over the lung not forming aggregates) in contrast to the other test groups and the control animals which occasionally had (multi)focal aggregates of macrophages. Alveolar macrophages of all treated animals had black, fibrous structures within their cytoplasm (regarded to be carbon nanotubes) (Figure 5B). On study day 25, this material was moved to the draining lymph node by alveolar macrophages (Figure 5F). The material was present intracellular (in macrophages) in 5 animals at 2.5 mg/m^3^ and in 2 animals exposed to 0.5 mg/m^3^.

#### Graphene

On study day 7, single macrophages or small aggregates of alveolar macrophages were observed in the lungs of all treated animals. Most of them were loaded with black variable sized particles (regarded as graphene). Most of these macrophages were located in the lumen of alveoli, few occurred in the alveolar wall, the alveolar ducts, and in terminal bronchioles. The accumulation of macrophages increased with the exposure concentration. In addition, single ‘microgranulomas’ were observed in animals exposed to 2.5 and 10 mg/m^3^ (Figure 5C). Microgranulomas were characterized by small particle-loaded aggregates of macrophages that partly appeared fused or showed single giant cells and were connected to the alveolar septum. They were not associated with any other inflammatory response and there were no further alterations of the lung parenchyma.

On study day 28, the occurrence of particle-loaded macrophages or aggregates of macrophages was still concentration-dependently increased. Incidence and severity were comparable to the groups examined on day 7. In addition, single or few microgranulomas were observed in animals exposed to 2.5 and 10 mg/m^3^. Two animals exposed to 10 mg/m^3^ showed single small particles in the mediastinal lymph nodes without any histopathological findings. One animal exposed to 0.5 mg/m^3^ showed macroscopically enlarged mediastinal lymph nodes with a moderate lympho-reticular cell hyperplasia. This was considered to be incidental.

#### Graphite nanoplatelets

On study day 4, the lungs of one animal exposed to 10 mg/m^3^ showed few intra-alveolar located multifocal aggregates of alveolar macrophages. All animals exposed to this concentration (10 mg/m^3^) showed black, irregularly shaped particles within the cytoplasm of single intra-alveolar macrophages (regarded as graphite nanoplatelets) (Figure 5D).

On study day 25, as well as on study day 95, particles were still observed in alveolar macrophages. Few intracellular particles in the mediastinal lymph node (within macrophages) were observed in one animal exposed to 10 mg/m^3^ on study day 95.

No compound-related microscopic alterations were observed after exposure to 0.5 and 2.5 mg/m^3^ Graphite nanoplatelets.

#### Carbon Black

On study day 4, black particles (regarded as Carbon Black) were observed within alveolar macrophages in animals exposed to 10 mg/m^3^. In three of the six treated animals there was a minimal increase in numbers of alveolar macrophages (Figure 5E). There were no other findings.

Among the four tested carbon-based nanomaterials, MWCNT caused the strongest effects in the lower respiratory tract (granulomas, inflammation). Most prominent changes in BALF cellularity comprised marked increases in polymorphonuclear neutrophils and lymphocytes, which decreased but were still present after 28 days. Markedly increased activities of MPO are in line with the observed increased presence of polymorphonuclear neutrophils.

Changes observed after inhalation of graphene were less pronounced. Like MWCNT granuloma formation was observed in the lower respiratory tract. Increases of polymorphonuclear neutrophils and lymphocytes in BALF were less pronounced when compared to MWCNT. The increase in osteopontin concentrations was higher compared to MWCNT exposure, probably indicating macrophage and lymphocyte activation. Increased osteopontin concentrations were still observed on study day 28.

Only local inflammation and no further alterations were observed after exposure to MWCNT or graphene.

Macrophage aggregates and phagocytized particles were the only microscopically visible alteration observed after inhalation of graphite nanoplatelets. Merely a slight, transient increase of GGT activity was present in BALF.

Neither adverse microscopic effects nor changes in broncho-alveolar parameters were observed after inhalation of Carbon Black.

## Discussion

All four carbon-based nanomaterials were generated at particle sizes distributions which did enable the vast majority of the particles to reach the lower part of the respiration tract in rats. When comparing the concentration at 2.5 mg/m^3^, MMADs were approximately 2 μm in case of graphite nanoplatelets, slightly below 2 μm in case of MWCNT, whereas they were in the submicron range in case of Carbon Black and graphene (0.6 μm and smaller than 0.4 μm, respectively).

However, regarding the observed toxicological effects, MWCNT produced the strongest response, even at a considerably higher MMAD and a 4-fold lower concentration compared to graphene and Carbon Black. Therefore, the reason for difference in observed toxicity appears not primarily driven by particle size.

**Figure 6:**
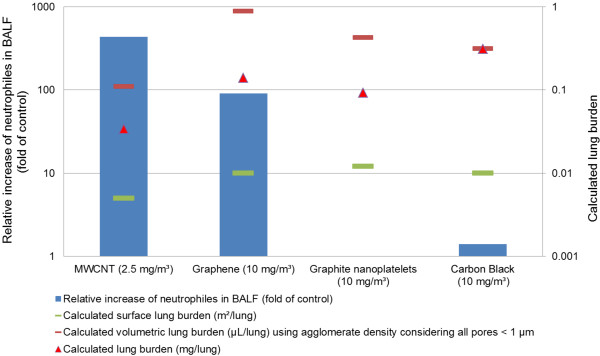
Correlation of polymorphonuclear neutrophils in BALF with calculated lung burden on study day 7.

Many factors determining the toxicity of nanomaterials have been discussed: Structural properties have been discussed as an important factor governing biological effects of nanomaterials, such as structural defects [32,33], fiber length [22,34], aggregation state [18,29,35-37], but also surface properties such as surface charge [38,39], hydrophilicity and oxidation state [40]. Another factor contributing to toxicity of inhaled nanomaterials (and other particles) is the impairment of alveolar clearance via macrophages. In case of otherwise ‘inert’ compounds, this process is triggered by volumetric overload of alveolar clearance capacity, in which the deposited volume is considered to be the most important parameter [41,42]. The deposited volume can only be derived by mass deposition divided by density. As mass deposition of carbon-based materials cannot be reliably determined, it was calculated using Multiple-Path Particle Dosimetry (MPPD) modeling software (Table 11). Surface lung burden was calculated by multiplying lung deposition derived by MPPD modeling software with specific surface area determined by Hg porosimetry. Concerning relationship between surface and toxicity, there was apparently no correlation at all, as the lowest surface burden led obviously to the most severe pulmonary effects. To derive the volumetric load, two approaches were used to approximate the 'true' agglomerate density. One used the apparent density, which was derived by measuring weight of a certain volume of test material without any manipulation. The apparent density is the lowest density of the respective material. It includes also large pores between the more dense packed agglomerates. The other approach included all pores < 1 μm as determined by mercury porosimetry, because the aerodynamic diameters of the agglomerates were mostly between 1 and 2 μm. Results of both approaches are presented in Table 11. However, effect and potency of the four tested carbon-based materials did not correlate well with mass lung load, volumetric lung load or surface lung load. These parameters do not explain the observed differences in toxicity (Figure 6). The considerations presented above are approaches to evaluate the relationship of toxicity and several physico-chemical parameters. It was based on scientifically sound assumptions and must be verified by further studies.

**Table 11:** Dosimetry and calculated surface and volume lung burden

	**MWCNT**	**Graphene**	**Graphite nanoplatelets**	**Carbon black**
Highest target concentration [mg/m^3^]	2.5	10	10	10
Hg surface area [m^2^/g]	161	131	74	32
Apparent density [g/mL]	0.15	0.04	0.02	0.52
Calculated deposition using apparent density [mg/lung]^*^	0.03	0.26	0.20	0.10
Calculated volumetric lung burden using apparent density [μL/lung]^**^	0.19	4.79	3.67	0.50
Agglomerate density [g/mL]	0.39	0.29	0.89	1.05
Calculated deposition using agglomerate density [mg/lung]^*^	0.03	0.30	0.11	0.33
Calculated volumetric lung burden using agglomerate density [μL/lung]^**^	0.07	1.02	0.52	0.31
Calculated surface lung burden using agglomerate density [m^2^/lung]	0.005	0.039	0.008	0.010
Pulmonary response	+++	+	(−)	-

* Calculated with Multiple-Path Particle Dosimetry Model (MPPD v 2.11).

** Based on calculated disposition data.

## Conclusion

Marked differences in toxicity were observed after short-term inhalation exposure of four carbon-based nanomaterials. Whereas no relevant toxicity occurred with Carbon Black and graphite nanoplatelets, MWCNT and graphene induced lung toxicity. The toxicity of MWCNT at 2.5 mg/m^3^ was more marked than that of graphene at 10 mg/m^3^. Neither differences in the mesoscopic structure of the nanomaterials (as measured by surface area, apparent density and agglomerate density) nor differences in the lung burden (surface or volume lung burden based on calculated lung deposition) correlated well with the differences in toxicity. Since the level of impurities was insufficient to induce the observed effects, either other factors (*e.g.* the different chemical reactivity at the accessible part of the nanomaterials) or complex combinations of the several parameters determine the toxic potency of the different carbon-based nanomaterials. Until the properties which govern the toxicity are identified, testing by short-term inhalation is the best option to identify hazardous properties in order to avoid unsafe applications or select safer alternatives for a given application.

## Methods

### General

The present studies were conducted according to the OECD Principles of Good Laboratory Practice [43], which principally meet the United States Environmental Protection Agency Good Laboratory Practice Standards [40] CFR Part 160 (FIFRA) and Part 792 (TSCA)]. The study was conducted referring to OECD Guideline 412 [44].

### Test materials and characterization

MWCNT (Graphistrength™ C100, purity >95%), was manufactured by Arkema (Lacq, France). Graphene was produced by thermal shocking of graphite oxide resulting in simultaneous exfoliation and chemical reduction. Graphite nanoplatelets were obtained by flash heating of intercalated graphite. Low surface Carbon Black (37 m^2^/g) was provided by Evonik-Degussa (Germany). The test materials were examined for the applicable physical-chemical endpoints, including representative images (SEM), particle size distribution (SEM) of both primary structure and agglomerates, crystallite size (X-ray diffraction (XRD)), crystalline phase (XRD), spectroscopy (Raman), surface chemistry (XPS) and secondary ion mass spectrometry (SIMS), dispersability in water and in serum-containing media (by analytical ultracentrifugation (AUC)), pore sizes (Hg intrusion) and the derived values of specific surface area, apparent density, and agglomerate density. Additionally, the materials were examined for endotoxin content using the chromogenic Limulus Amebocyte Lysate Kinetic-QCL Test [45]. No endotoxins were detected in MWCNTs, Graphene, and Graphite Nanoplatelets, and only a negligible endotoxin content was measured in Carbon Black (0.05 EU/mL).

### Animals

Permission for animal studies was obtained from the local regulatory agencies, and all protocols were in compliance with the federal guidelines. The studies were performed in an AAALAC-approved laboratory in accordance with the German Animal Welfare Act and the effective European Council Directive. Male Wistar (strain Crl:WI (Han)) rats (7 weeks of age) were obtained from Charles River Laboratories (Sandhofer Weg, Sulzfeld, Germany) and were allowed free access to mouse/rat laboratory diet (Provimi Kliba SA, Kaiseraugst, Switzerland) and water. The animals were housed singly in mesh floored cages in accommodation maintained at 20 to 24°C, with a relative humidity of 30 to 70%, a light/dark cycle of 06.00 to 18.00 h light and 18.00 to 06.00 h dark and were allowed to acclimatize to these conditions for approximately two weeks before commencement of the study.

### Exposure regimen/test groups

Study design was similar in all experiments, but different in details as outlined below.

#### MWCNT and Carbon Black

Groups of 11 male Wistar rats were head-nose exposed to respirable dusts on 6 hours per day, on 5 consecutive days (days 0 to 4). The target concentrations were 0.1, 0.5, or 2.5 mg/m^3^ (MWCNT), or 0.5, 2.5, or 10 mg/m^3^ (Carbon Black). A concurrent control group was exposed to conditioned air. On study day 4 (after the last exposure) and 25 (21 days after the last exposure), 6 animals per group were sacrificed and designated for histopathological examinations. On study day 7 (3 days after last exposure) and 28 (24 days after last exposure), the remaining 5 animals per group were sacrificed. The lungs of these animals were lavaged, and BALF was analyzed for markers indicative for injury of the bronchoalveolar region.

#### Graphene

Groups of 8 male Wistar rats were exposed to target concentrations of 0.5, 2.5, or 10 mg/m^3^ or to conditioned air for 6 hours per day for 5 consecutive days (days 0 to 4). Animals were sacrificed on study day 7 and 28. On each sacrificing day, 3 animals per group were designated for histopathological examination and the remaining 5 animals per group for bronchoalveolar lavage. Sampling for histopathological examination was performed three days later compared to the other materials due to logistical reasons.

#### Graphite nanoplatelets

Groups of 8 male Wistar rats were exposed to 0.5, 2.5, or 10 mg/m^3^ or to conditioned air for 6 hours per day for 5 consecutive days (days 0 to 4). Animals designated for histopathological examinations were sacrificed on study day 4, 25 (only controls and 10 mg/m^3^), and 95. BALF was collected on study day 7, 28, and 98. On each sacrificing day, 3 animals per group were designated for histopathological examination and 5 animals per group for bronchoalveolar lavage.

### Generation of the test atmospheres

Brush dust generators (developed by the Technical University of Karlsruhe in cooperation with BASF, Germany) served for generation of test atmospheres with MWCNT, graphite nanoplatelets, and Carbon Black. In case of graphene, test atmospheres were produced with swinging bed dust generators (in house development by BASF), since it was not possible to generate adequate aerosols of graphene with the brush dust generator.

Generated dusts were mixed with compressed air (filtered air pressurized to about 6 bar, flow rate 1.5 ± 0.3 m^3^/h) in a glass tube, diluted with conditioned air (activated charcoal-filtered air, 22 ±2°C, 50 ± 20% relative humidity, flow rate 4.5 ± 0.3 m^3^/h) and passed via a cyclone into the inhalation system. The 90-l cylindrical stainless steel inhalation chamber was fed via a cone-shaped inlet at the top and exhausted at the opposite end. The desired inhalation chamber concentrations were achieved by withdrawing/exhausting and replacing a portion of the dust aerosol air with conditioned supply air immediately before entering the chamber (6 m^3^/h). Mean flow rate through the inhalation chamber, measured at exhaust air, was 5.4 ± 0.3 m^3^/h for all concentrations, that is, air was changed in the inhalation chambers about 67 times per hour.

**Figure 7:**
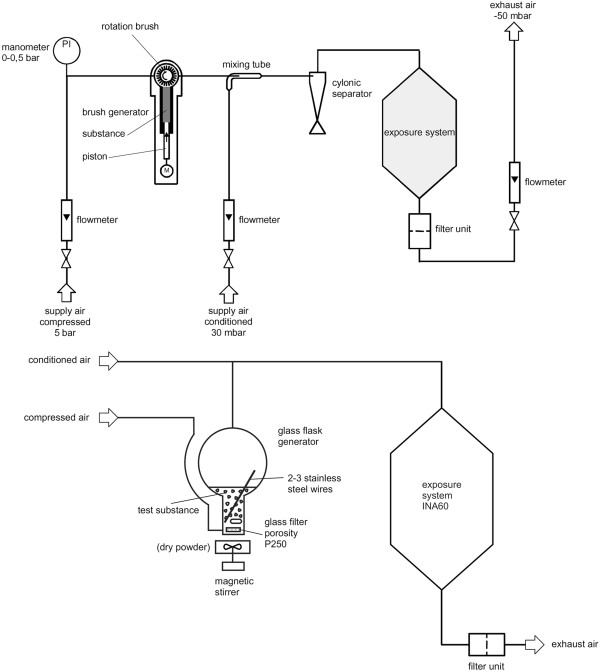
**Schematic picture of the Inhalation exposure system.** Aerosols of MWCNT, graphite nanoplatelets, and Carbon Black were generated with a brush generator (top); graphene was dispersed via a swinging bed generator (bottom).

A schematic diagram of the inhalation system is shown in Figure 7.

### Monitoring and characterization of the test atmosphere

Compressed and conditioned supply air and exhaust air flow rates, chamber temperature and humidity were measured automatically with appropriate sensors/orifice plates; data were saved every 10 s and retained for analysis. To ensure the stability of the dust aerosols, the inhalation chambers were monitored continuously during exposure using scattered light photometers (VisGuard; Sigrist-Photometer AG, Switzerland; for details see [27]). To quantify the atmospheric dust concentration, gravimetric measurements of air samples taken adjacent to the animals’ breathing zone were performed (probe internal diameter 7 mm). A defined volume of sample air was drawn by vacuum pump across a binder-free glass-fiber filter paper (Macherey-Nagel MN 85/90 BF, diameter 4.7 cm).

Aerosol dust concentration was calculated as the increase in weight of the filter after sampling, divided by sample volume at test conditions (22°C, atmospheric pressure, 50% relative humidity). As a rule, two samples were taken per exposure and concentration group. The duration of sampling was adjusted to the test substance concentration in the chamber to obtain a total sample weight of 1 to 5 mg. Thus, the volume of the air samples varied with the atmospheric concentration. To determine the MMAD (the calculated aerodynamic diameter which divides the size distribution in half when measured by mass), cascade impactor measurements were performed with a Sierra Marple 298 cascade impactor. The effective aerodynamic cut-off diameters were 21, 15, 10, 6.5, 3.5, 1, 0.7, and 0.4 μm. To capture the particles < 0.4 μm, the impactor was equipped with a backup filter. The deposition on each impactor stage as well as on the backup filter was determined gravimetrically. Particle size distributions were calculated according to DIN 66141 and DIN 66161, i.e. linear regression of cumulative percent (probit values) *versus* logarithms of effective cut-off diameters. Particle size distributions measured by cascade impactor were expressed as MMAD and geometrical standard deviation (GSD). Additionally, a light-scattering spectrometer (WELAS 2100; Palas, Karlsruhe, Germany) was used for particle sizes from 0.24 to 10 μm (at least 10 repeats). In the submicrometer range (11 to 1083 nm), particle size distribution was measured with a Scanning Mobility Particle Sizer equipped with a condensation particle counter (SMPS + C) (Grimm Aerosol Technik GmbH, Ainring, Germany). Particles were classified by electrostatic fractionation of the different sized particles. Particle counts in each of the fractions were counted by condensation particle counter. The SMPS spectrometer uses electrical mobility to measure the particle size. This technique utilizes a bipolar charger to impact a known charge distribution on the aerosol sample, and classify particles according to their ability to traverse an electric field. The data are interpreted based on a spherical particle model. While this method is appropriate for sizing spherical particles, it leads to errors in the mobility size data interpretation for agglomerates and aggregates. As the aerosols examined in this paper are mainly irregular agglomerates consisting of either tubes or sheets, the relevance of using SMPS is somewhat limited.

Additionally, samples of the test atmospheres of MWCNTs, graphene and graphite nanoplatelets were collected using gold-coated capillary filters and evaluated by scanning electron microscopy. In case of MWCNTs, the test atmosphere was generated under the same conditions as in the toxicity study at a later time point.

### Animal exposure

During exposure, rats were restrained in glass tubes fixed to the inhalation chamber walls with their snouts projecting into the inhalation chamber (head/nose exposure). Overpressure was maintained inside the inhalation chamber to ensure that the aerosol in the animals’ breathing zone was not diluted by laboratory air. The exposure systems were kept under exhaust hoods in an air-conditioned room.

### Clinical observations

Health status and cage-side clinical signs were checked at least once daily (on exposure days before, during and after exposure). Body weights were measured weekly.

### Hematology and acute phase proteins

Before sacrifice, blood samples for hematology and clinical chemistry were taken from all animals designated for collection of BALF by retrobulbar venous plexus puncture under isoflurane (Isoba®, Essex GmbH Munich, Germany) anesthesia. Hematology (ADVIA120 Instrument, Siemens, Germany) comprised red blood cell counts, hemoglobin, hematocrit, mean corpuscular volume, mean corpuscular hemoglobin content, mean corpuscular hemoglobin concentration, platelets, total white blood cell and differential blood cell counts.

Acute phase proteins: rat α_2_-macroglobulin was measured with an MTP-ELISA (Kamiya Biomedical Company, Seattle, USA, cat no. KT-353), measured with a Sunrise MTP Reader, Tecan AG, Switzerland, by using the Magellan Software provided by the instrument producer. Haptoglobin was measured photometrically based on the preservation of the hemoglobin peroxidase activity (Tridelta Ltd, Maynooth, Ireland, cat no. TP-801), running on a Hitachi 917 instrument, Roche, Basel Switzerland.

### Broncho-alveolar lavage and lung tissue homogenate

To obtain BALF, animals were killed by exsanguination under Narcoren® anesthesia and the lungs lavaged twice with 6 ml (22 ml/kg body weight) physiological saline. The two washes were combined (an average of 11 ml of lavage fluid was recovered per animal) and aliquots of the combined washes were used for the determination of cytology, total protein concentration and enzyme activities, as well as mediators.

Total BALF cell counts were determined with an Advia 120 (Siemens Diagnostics, Fernwald, Germany) haematology analyzer. Counts of macrophages, polymorphonuclear neutrophils, lymphocytes, eosinophils, monocytes and atypical cells were performed on Wright-stained cytocentrifuge slide preparations as described by Warheit and Hartsky [46]. The differential cell count was evaluated manually by counting at least 400 BALF cells per sample. The following parameters were measured with a Hitachi 917 (Roche Diagnostics, Mannheim, Germany) reaction rate analyzer: total protein (turbidimetric method with Benzethonium chloride), LDH (EC 1.1.1.27; kinetic UV test, 340 nm, 37°C acc. to IFCC), ALP (EC 3.1.3.1; kinetic colour test, 450 nm, 37°C acc. to IFCC), NAG (EC 3.2.1.30; colour test, 580 nm, 37°C) [47] and GGT (EC 2.3.2.2, Szasz method) (kinetic colour test, 415 nm, 37°C acc. to IFCC) activities.

In case of MWCNT mediators in BALF were measured at Rules-based Medicine Inc., Austin, TX, USA, with xMAP technology (Luminex Corp., Austin, TX, USA) as described previously [48-51]. These parameters comprised various cytokines, chemokines, adhesion molecules, matrix metalloproteinases, acute phase proteins, signal proteins of apoptosis or cell proliferation: apolipoprotein A1, β-2 microglobulin, calbindin, CD40, CD40L, clusterin, C-reactive protein, cystatin C, epidermal growth factor (EGF), endothelin-1, eotaxin, factor VII, fibroblast growth factor (FGF)-basic, FGF-9, fibrinogen, GCP-2, granulocyte-macrophage colony-stimulating factor (GM-CSF), growth hormone, glutathione-S-transferase (GST)-α, GST-1 Yb, haptoglobin, interferon (IFN)-γ, IgA, IL-1α, IL-1β, IL-2, IL-3, IL-4, IL-5, IL-6, IL-7, IL-10, IL-11, IL-12p70, IL-17, insulin, interferon gamma-induced protein 10 (IP-10), KC/GROα, leptin, leukemia inhibitory factor (LIF), lymphotactin, MCP-1, MCP-3, MCP-5, M-CSF, macrophage-derived chemokine (MDC), MIP-1α, MIP-1β, MIP-1γ, MIP-2, MIP-3β, matrix metalloproteinase-9 (MMP-9), myoglobin, MPO, NGAL, oncostatin M (OSM), osteopontin, regulated on activation, normal T cell expressed and secreted (RANTES), stem cell factor (SCF), serum amyloid P, serum glutamic oxaloacetic transaminase (SGOT), tissue inhibitor of metalloproteinase-1 (TIMP-1), tissue factor, tumor necrosis factor α (TNF-α), thrombopoietin (TPO), vascular cell adhesion molecule-1 (VCAM-1), VEGF, and van Willebrand factor. Additionally, the cytokine-induced neutrophil chemoattractant-1/IL-8 (CINC-1/IL-8) levels in BALF and lung tissue homogenates were measured with an ELISA produced by R&D Systems Inc., Minneapolis, US, (Quantikine rat CINC-1, cat. no. RCN100) by using a Sunrise MTP Reader, Tecan AG, Switzerland, with the Magellan Software provided by the instrument producer. The mediator level changes were regarded relevant when their levels were above the detection limit in controls and/or dose group samples and when they were at least increased above 2-fold in the (high) dose group. Based on these results and taken into account previous data of the whole panel of mediators the following parameters were considered to be most suitable for characterizing of lung inflammation and were measured in case of graphene, graphite nanoplatelets and Carbon Black: rat monocyte chemoattractant protein-1 level (rat MCP-1); instant ELISA, Bender MedSystems, Vienna, Austria (cat. no BMS631INST); rat CINC-1/IL-8 level (ELISA, R&D Systems Inc., Minneapolis, US), (Quantikine rat CINC-1, cat. no. RCN100); macrophage colony stimulating factor (M-CSF; Quantikine Mouse M-CSF ELISA, R&D Systems Inc., Minneapolis, USA) (cat no. MMC00); rodent osteopontin; ELISA, R&D Systems, Inc., Minneapolis, US (Quantikine mouse osteopontin, cat. no. MOST00). The mediators were measured at a Sunrise MTP Reader, Tecan AG, Switzerland, by using the Magellan Software provided by the instrument producer. The monitoring panel was arranged so that different functional groups of mediators were covered, i.e., CC-chemokines (MCP-1); CXC-chemokines (IL-8/CINC-1); hematopoiesis (M-CSF); proliferation of sessile cells (osteopontin).

In order to evaluate if mediators were not washed out into the BALF (i.e. remained in the lung), the parameters were measured also in lung tissue homogenates: After bronchoalveolar lavage was performed the right lung portion of each animal was resected and stored at −80°C until lung tissue homogenate preparation: 0.2 g of the main lobe (lobus caudalis dexter) was mixed with 0.8 ml ice-cold Tissue Protein Extraction Reagent (T-PER, cat. no. 78510, Pierce Biotechnology, Rockford, IL, USA) added with a Complete Protease Inhibitor Cocktail (cat no., 11 873 580 001, Roche, Basel, Switzerland), homogenized for up to 40 seconds with an Ultra-Turrax (IKA, Staufen, Germany), and the homogenate centrifuged at 14,000 g and 4°C for 5 min.

In case of MWCNT, mediators in lung tissue homogenate were measured at Rules-based Medicine Inc., Austin, TX, USA, with xMAP technology (Luminex Corp., Austin, TX, USA) as mentioned above. The mediator level changes were regarded relevant when their levels were above the detection limit in controls and/or dose group samples and when they were at least increased above 2-fold in the (high) dose group. Comparing the mediator levels in BALF and lung tissue homogenates, together with results of previous studies, revealed that two mediators (IL-1α and TNFα) were consistently more pronounced increased in the lung tissue. Therefore, both parameters (cytokines indicating local inflammation) were used for monitoring in the studies with Graphene, Graphite Nanoplatelets and Carbon Black: rat IL-1α; FlowCytomix Rat IL-1α Simplex Kit; Bender MedSystems, Vienna, Austria (cat. no. BMS8627FF). The measurement was performed at the FACS Calibur flow cytometer, Becton Dickinson, Heidelberg, Germany and the evaluation was made with the FlowCytomix Pro Software, vs. 2.3, Bender MedSystems, Vienna, Austria; rat tumor necrosis factor-alpha (rat TNFα); Quantikine rat TNFα/TNFSF1A ELISA; R&D Systems Inc., Minneapolis, US, (cat. no. RTA100). The measurements were performed with a Sunrise MTP Reader, Tecan AG, Switzerland, by using the Magellan Software provided by the instrument producer.

### Pathology

Animals were euthanized by exsanguination under Narcoren® anesthesia. Gross necropsy was carried out. Lungs were instilled at a pressure of 20 to 30 cm of water. Weights of brain with olfactory bulb, lungs, and mediastinal lymph nodes were determined.

Brain, head (with oropharynx), larynx, lungs, mediastinal lymph nodes, and trachea were fixed in 4% buffered formaldehyde (corresponding to 10% formalin), paraffin embedded, sectioned and stained with hematoxylin-eosin for histopathology.

Light microscopic examination was performed on the respiratory tract comprising nasal cavity (four levels), larynx (three levels), trachea (longitudinal with carina), lung (five lobes), and mediastinal lymph nodes.

### Statistical analysis

Dunnett’s test [52,53] was used for simultaneous comparison of all concentration groups with the control group for body weights and body weight changes. Clinical pathology parameters were analyzed by non-parametric one-way analysis using the Kruskal-Wallis test (two-sided). If the resulting p-value was equal or less than 0.05, a pair-wise comparison of each dose group with the control group was performed using Wilcoxon-test or Mann–Whitney-U-test (two-sided) for the equal medians. Statistical significance was defined as p ≤ 0.05 compared with the control group [54]. Organ weights were compared among groups by nonparametric one-way analysis using the two-sided Kruskal–Wallis test, followed by a two-sided Wilcoxon test for the hypothesis of equal medians in case of p ≤ 0.05 [55-57].

## Abbreviations

ALP: Alkaline phosphatase; AUC: Analytical ultracentrifugation; BALF: Broncho-alveolar lavage fluid; CINC: Cytokine-induced neutrophil chemoattractant; CNT: Carbon nanotubes; EGF: Epidermal growth factor; FGF: Fibroblast growth factor; GCP: Granulocyte chemotactic peptide; GM-CSF: Granulocyte-macrophage colony-stimulating factor; GGT: γ-Glutamyl-transpeptidase; GRO: Growth related oncogen; GST: Glutathione-S-transferase; ICP-MS: Inductively coupled plasma optical emission spectrometry; IFN: Interferon; IL: Interleukin; IP-10: Interferon gamma-induced protein 10; LDH: Lactate dehydrogenase; LIF: Leukemia inhibitory factor; M-CSF: Macrophage colony stimulating factor; MCP: Monocyte chemoattractant protein; MDC: Macrophage-derived chemokine; MIP: Macrophage inflammatory protein; MMAD: Mass median aerodynamic diameter; MMP: Matrix metalloproteinase; MPPD: Multiple-path particle dosimetry model; MPO: Myeloperoxidase; MWCNT: Multi-walled carbon nanotubes; NAG: N-Acetyl-β-glucosaminidase; NGAL: neutrophil gelatinase-associated lipocalin; OPC: Optical particle counter; OSM: Oncostatin; RANTES: Regulated on activation, normal T cell expressed and secreted; SCF: Stem cell factor; SEM: Scanning electron microscopy; SGD: Geometric standard deviation; SGOT: Serum glutamic oxaloacetic transaminase; SIMS: Secondary ion mass spectrometry; SMPS: Scanning mobility particle sizer; SWCNT: Single-walled carbon nanotubes; TIMP: Tissue inhibitor of metalloproteinase; TNF: Tumor necrosis factor; TPO: Thrombopoietin; VCAM: Vascular cell adhesion molecule; VEGF: Vascular endothelial growth factor; XPS: X-ray photoelectron spectroscopy; XRD: X-ray diffraction.

## Competing interests

The authors are employees of BASF SE, a company producing and marketing nanomaterials.

## Authors’ contributions

LM carried out the inhalation experiments. VS performed the analysis of BALF. ST, KK, and SG carried out the histopathological evaluation. WW performed the physico-chemical characterization of the materials. TH is the author of the manuscript. KW, BR, and RL contributed to the conceptual design of the studies and interpretation of the results. All authors read and approved the final manuscript.
